# Psychometric Examination of the Body, Eating, and Exercise Comparison Orientation Measure (BEECOM) among Spanish Adolescents and Young Adults

**DOI:** 10.3390/nu15030626

**Published:** 2023-01-26

**Authors:** Adrian Paterna, Manuel Alcaraz-Ibáñez, Alvaro Sicilia

**Affiliations:** Health Research Centre and Department of Education, University of Almería, 04120 Almería, Spain

**Keywords:** eating disorders, eating pathologies, disordered eating, social comparison, body image, psychometrics, factorial validity

## Abstract

The Body, Eating, and Exercise Comparison Orientation Measure (BEECOM) has been frequently used within the context of research on eating disorders (ED). Although both long (BEECOM-L) and short (BEECOM-S) versions of the instrument exist, their psychometric properties have not yet been concurrently investigated across different populations in terms of age and gender. The present study aimed to compare the psychometric properties of both the BEECOM-L and the BEECOM-S among Spanish male and female non-clinical adolescents and young adults. Data from 4 samples including 1213 middle school and college students enrolled in 10 education centers from southern Spain (age ranging from 12 to 35 years, *M*_age_ = 17.796, *SD*_age_ = 4.796, 53% females) were analyzed using factorial, correlation, and regression analysis techniques. Results provided evidence that support the reliability, measurement invariance according to age and gender, and convergent/incremental validity for the scores from both the BEECOM-L and BEECOM-S. Concerning factorial validity, marginally acceptable and adequate goodness-of-fit indices were obtained for the BEECOM-L and BEECOM-S, respectively. The BEECOM-S proves to be a psychometrically sound instrument with potential value for assessing social comparisons focused on body, eating, and exercise in non-clinical adolescents and young adults from Spain.

## 1. Introduction

Eating disorders (ED) are a group of increasingly prevalent psychiatric pathologies that, having a relative bad prognosis, account for significant morbidity and mortality in Western countries [1,2,3]. In this vein, the fact that around a third of women and half of men who meet the clinical criteria for ED do not seek professional help [4] means that the social and economic burden of ED extends beyond healthcare costs [5]. As a result, identifying modifiable risk factors for ED on which to focus treatment and prevention efforts has been a major research priority [6,7,8].

A cognitive process that has been consistently identified as a modifiable risk factor for ED is the tendency towards body/appearance-related social comparison [9,10,11,12]. However, an emerging line of research has begun to demonstrate that further consideration of comparisons focused on actions aimed at improving body appearance, such as eating or exercise behavior, may provide deeper insight into the cognitive processes underlying ED [13,14,15,16]. This body of research has been built around the only self-reported quantitative measure proposed to date for the purpose of assessing the three facets of ED-related social comparisons outlined above (i.e., the Body, Eating, and Exercise Comparison Orientation Measure, BEECOM) [17].

Findings from several research studies have been consistent in supporting the psychometric properties of the BEECOM scores in terms of both reliability and convergent/incremental validity [14,16,17,18]. In contrast, the available evidence on the factorial validity of the instrument is far less conclusive. For example, evidence has been provided that supports the original long 18-item structure consisting of 6 items each for the three lower-order factors (body-, eating-, and exercise-related comparison orientation) and a higher-order factor (ED-related comparison orientation) on a sample of non-clinical U.S. college females [17]. However, this factor structure is not supported by the results of two subsequent studies. Firstly, the one conducted in a non-clinical sample of Iranian university male and female students with the aim of providing a Farsi translation of the original long version of the BEECOM (hereinafter BEECOM-L) revealed gender differences in the distribution of several items across the lower-order factors [18]. Secondly, the one conducted on a sample of U.S. females in their young adulthood revealed that several items did not load clearly on their theoretically relevant lower-order factors in the clinical subsample in terms of ED [16]. This finding led the authors to propose a short version of the instrument (BEECOM-S) consisting of 9 items (3 for each lower-order factor) instead of 18 items, whose adequate fit was subsequently corroborated in a second non-clinical subsample of otherwise similar socio-demographic characteristics to the first one [16].

Taken together, the available evidence suggests that (i) the content of BEECOM might be subject to gender-specific interpretations, which would make this instrument unsuitable for making comparisons across groups based on this variable and (ii) that the revised short version of the instrument is likely to outperform the original long version in psychometric terms. However, the limited number of studies from which such evidence is drawn, coupled with the particular socio-demographic profiles of the participants included in these studies, precludes generalizing the above conclusions to other populations of interest in the context of ED. A clear example of such a population would be adolescent girls and boys under 17 (the minimum age of individuals on which the psychometric properties of the BEECOM have been tested so far [17]), who represent an important target group for ED prevention efforts [19]. The latter is clear in the light of the evidence suggesting that (i) 25–50% of ED onset occurs before the age of 17 years and (ii) that the differences in prevalence levels traditionally observed in favor of females are decreasing [4].

Simultaneous examination of the psychometric properties of both versions of the BEECOM in a wider range of populations in terms of age and gender than hitherto considered may provide valuable information on the applicability of the instrument and, in particular, with regard to the comparative psychometric performance of the two versions. Thus, should evidence confirming the superior performance of the short version be obtained, then its use could be recommended. This would mean halving the application time of the BEECOM, which may decrease the response burden and increase the response rate of surveys including this instrument [20]. In addition, evidence derived from examining whether the BEECOM scores are invariant across populations consisting of individuals of different gender and age would allow recommendations to be made concerning the appropriateness of using these scores in two main situations: firstly to make reasonably unbiased comparisons between these population groups [21] and secondly in the context of conducting regression analysis involving moderation testing, which—as a result of considering group membership as the moderating variable instead of conducting subgroup analyses—would benefit from an increased level of statistical power [22].

In view of the above, the main objective of the present study was to compare the psychometric properties of the scores derived from the BEECOM-L and the BEECOM-S in terms of factorial validity, reliability, measurement invariance according to age and gender, and convergent/incremental validity. Given the paucity of research on the topic and the inconclusive nature of the available evidence, no hypothesis was advanced concerning which version of the instrument would show better overall psychometric performance. According to findings from previous research, it was hypothesized that the BEECOM scores would show high levels of reliability in terms of internal consistency (*α*) or construct reliability (ρ) (i.e., >0.80) [14,17,18]. It was also expected that, providing evidence of convergent validity, the BEECOM scores would be positively correlated with two main groups of variables: firstly with comparisons strictly focused on physical appearance, with effect sizes expected to fall within a range of *r* = 0.53 to *r* = 0.76) [17] and secondly with several ED outcomes, these including (i) overall ED symptoms, with effect sizes expected to fall within a range of *r* = 0.37 to *r* = 0.71) [16,18]; (ii) weight and shape concerns, with effect sizes expected to fall within a range of *r* = 0.38 to *r* = 0.77) [13,17,23]; and (iii) dietary restraint, with effect sizes expected to fall within a range of *r* = 0.20 to *r* = 0.46) [13,18]. Finally, it was hypothesized that, consistent with the results of previous research providing evidence of incremental validity for the BEECOM scores [16,17], these would account for additional variance in all three ED outcomes under consideration over and above that accounted for by both body mass index (BMI) and the scores derived from a measure strictly focused on appearance-related comparisons. In the event that evidence was obtained to support measurement invariance of the BEECOM scores across age and gender groups, a secondary goal of the present study was to examine gender and age differences in ED-related comparison orientation. It was hypothesized that, in line with previous research examining appearance-focused comparisons [10,12,24], small-to-intermediate-sized differences would be found in favor of groups consisting of females and younger adults.

## 2. Materials and Methods

### 2.1. Sample Size Calculation

The sample size of the study was drawn using two different procedures. Firstly, a Monte Carlo simulation was conducted in Mplus version 7 (http://www.statmodel.com/index.shtml, accessed on 21 December 2022) [25] to determine the sample size needed to obtain reliable estimations for the parameters involved in the confirmatory factor analyses (CFA) [26]. This simulation was conducted taking into account the main features of the most complex model (i.e., the one corresponding to the BECCOM-L) in terms of the number of factors/indicators, the mean values expected for factor loadings, and the possible absence of normality of the data. The results of this simulation suggested that, assuming a missing data value of 1%, a sample size of 250 participants per group was sufficient to provide 80% statistical power in the estimation of the model parameters. Secondly, a power analysis using G*Power version 3.1 (Heinrich Heine University Düsseldorf, Düsseldorf, Germany) [27] was conducted to determine the sample size needed to conduct the regression analyses. The results suggested that that 1021 participants were needed to detect small effect sizes (i.e., *f*^2^ = 0.025; α = 0.05 two tailed; 95% power) in regression models involving 11 predictors.

### 2.2. Participants

A total of 1297 high school, middle school, and college students enrolled in 10 different education centers from southern Spain were invited to participate in the study using a non-probabilistic sampling technique. The following inclusion criteria were applied: (i) for the older age group, having a similar age to that of the participants included in the studies in which the BEECOM-L and the BEECOM-S were proposed (i.e., between 17 and 35 years old) [16,17]; (ii) for the younger age group, being aged between 12 and 16 years old; and (iii) for both age groups, to provide informed consent to participate in the research, which was additionally requested from parents or legal guardians for individuals aged under 18 years old. Failure to provide informed consent either by themselves (*n* = 18) or by parents or legal guardians (*n* = 29) led to the exclusion of 47 potential participants. Data were excluded from the analyses when the participants (i) reported having a clinical diagnosis in terms of ED (*n* = 10); (ii) identified themselves as non-binary in terms of gender (*n* = 14); or (iii) failed the embedded validity check question (*n* = 13). Thus, data from 1213 participants who mostly identified themselves in terms of ethnicity as White/Caucasian (96%) were analyzed considering the following four subgroups: (i) younger females (*n* = 287; *M*_age_ = 14.066, *SD*_age_ = 1.262); (ii) older females (*n* = 360; *M*_age_ = 21.369, *SD*_age_ = 3.490); (iii) younger males (*n* = 295; *M*_age_ = 14.190, *SD*_age_ = 1.127); and (iv) older males (*n* = 271; *M*_age_ = 20.926, *SD*_age_ = 3.845).

### 2.3. Instruments

#### 2.3.1. Body, Eating, and Exercise Comparison Orientation Measure (BEECOM)

We used our Spanish translation (see File S1) of the BEECOM [17]. The 18 items comprising the instrument are rated on a 7-point scale from 1 (never) to 7 (always). The items are grouped into 3 factors consisting of 6 items (long version) or 3 items (short version) that assess body comparisons (e.g., “I compare my body shape to that of my peers”), eating comparisons (e.g., “During meals, I compare what I am eating to what others are eating”), and exercise comparisons (e.g., “I pay close attention when I hear peers talking about exercise in order to determine if I am exercising as much as they are”). Higher scores imply higher frequency of body, eating, and exercise comparisons. Values of internal consistency (*α*) and composite reliability (ρ) ≥ 0.875 and 0.876 (BEECOM-L), and ≥0.807 and 0.813 (BEECOM-S) were respectively found in the present study (see Table 1 for details).

#### 2.3.2. Physical Appearance Comparison Scale-Revised (PACS-R)

We used a Spanish translation [10] of the PACS-R [28]. The 11 items comprising the instrument (e.g., “When I’m out in public, I compare my body size to the body size of others”) are rated on a 5-point scale from 0 (Never) to 4 (Always). Higher scores imply higher frequency of physical appearance comparisons. Evidence has been found that supports the validity and reliability of the scores of this instrument in both male and female individuals from Spain [10,12]. Values ≥ 0.943 (*α*) and 0.962 (ρ) were found for the total score of the PACS-R in the present study (see Table 1 for details).

#### 2.3.3. Eating Disorder Examination—Questionnaire Short (EDE-QS)

We used a Spanish translation [29,30] of the EDE-Q [31], which incorporated the two main modifications proposed in the short version of the instrument (i.e., a recall period on 7 instead of 28 days and 4 instead of 7 response categories) [32]. The 12 items comprising the instrument (e.g., “Have you been deliberately trying to limit the amount of food you eat to influence your weight or shape [whether or not you have succeeded]”), are rated on a 4-point scale from 0 (0 days or Not at all) to 3 (6–7 days or Markedly) and cover essential symptoms of anorexia nervosa, bulimia nervosa, and binge eating disorder [32]. According to the purposes of the present study, and consistent with previous research, three different scores derived from the instrument were used: (i) a score reflecting overall ED symptomatology [14,16,17,18]; (ii) an aggregate score of items covering dietary restraint symptoms (i.e., Items 1 and 2) [13,16,18]; and (iii) an aggregate score of items covering weight and shape concerns (i.e., Items 4, 5, and 6) [13,17,23,33]. Values ≥ 0.864 (α) and 0.932 (ρ) were found for the total scores of the EDE-QS in the present study (see Table 1 for details).

#### 2.3.4. Demographics

Demographic data concerning date of birth, gender, ethnicity, current diagnosis of ED, and self-reported height and weight were collected via a set of questions created for the present study. Height and weight were employed to calculate BMI (kg/m^2^). Given that the study sample consisted of both adolescents and adults, BMI values were subsequently adjusted according to percentile distribution taking into account age and gender norms [34].

### 2.4. Translation and Procedure

Firstly, a Spanish version of the BEECOM was obtained by following the procedures recommended for conducting linguistic adaptation of instruments addressing constructs in the body image domain [35]. In brief, the procedure involved the following steps: (i) two translators independently forward-translated the BEECOM items from English to Spanish; (ii) the two translations were checked by a third independent translator who, after resolving the observed discrepancies, proposed a synthesized translation; (iii) the resulting Spanish version was back-translated into English by a fourth independent translator; (iv) the forward- and back-translations were examined by a committee comprising all the aforementioned translators and the first author of the present study, who agreed on the pre-final version; (v) this version was pilot-tested in a sample of 12 participants (*M*_age_ = 17.692, *SD*_age_ = 3.750; 50% females); none of them referred to the presence of items that, in their opinion, were ambiguous or difficult to understand.

Following approval by the ethics committee of the first author’s institution, potential participants were invited to participate in the study in classroom settings by one of the authors of the present study. The research project was described as a study on body, eating, and exercise attitudes. In the case of minors, prior consent was required from parents or legal guardians to participate in the research. After being informed of the voluntary, anonymous, and non-rewarded nature of their participation, those who provided their informed consent (96.43%) completed a paper-and-pencil questionnaire in the classroom setting, which took approximately 5–10 min to complete. The assessment instruments included in the survey were counterbalanced using two different arrangements. Upon completion of the questionnaire, the participants were thanked for their cooperation and then debriefed about the precise aim of the study. Data were collected within the first academic quarter of the 2019–2020 school year.

### 2.5. Analytic Plan

Firstly, the factor structure of the long and the short versions of the BEECOM was examined across the population subgroups of interest using CFA techniques in Mplus version 7. These analyses were conducted using a full information maximum likelihood estimation method with robust standard errors (MLR). Two main considerations were taken into account while deciding to use this estimator: (i) that the response format of the instrument (i.e., a 7-point Likert-type) allowed their scores to be safely treated as continuous [36] and (ii) that MLR was the estimation method used in the previous studies investigating the factor structure of the BEECOM [16,17]. As model fit indices, values ≥0.95 or ≥0.90 for the comparative fit index (CFI), and ≤0.06 or ≤0.08 for both the root-mean-square error of approximation (RMSEA) and the standardized root-mean-square residual (SRMR), respectively, are considered as excellent or marginally acceptable [37]. Potential sources of model misspecification were identified by examining modification indices (MI) [37]. Results derived from the CFA were employed for computing composite reliability [38], which—along with the internal consistency values—served to provide evidence on reliability of both versions of the BEECOM.

Secondly, examination of the measurement invariance of the BEECOM scores across the population subgroups was conducted using multigroup CFA following the procedure described elsewhere [21]. This involved testing the following six progressively constrained models: Model 0 (or baseline model), which examined the equivalence of the factor structure based on the simultaneous free estimation of the parameters in all the subgroups of interest; Model 1, which additionally examined the equivalence of the factor loadings of the three first-order factors; Model 2, which additionally tested the equivalence of the three factor loadings of the higher-order factor; Model 3, which additionally tested the equivalence of the items’ intercepts; Model 4, which additionally tested the equivalence of the intercepts of the three factor loadings of the higher-order factor; Model 5, which additionally examined the equivalence of the first-order factor disturbances; and Model 6, which additionally tested the equivalence of the item residual variances (i.e., the error variance of each item) [21]. Given that the Satorra–Bentler scaled chi-square test of model fit difference with MLR estimators is particularly sensitive to sample size and the absence of normality, evaluation of changes (∆) in CFI (<−0.010 signals lack of invariance) was alternatively considered [39]. In the presence of evidence supporting the invariant nature of the BEECOM scores across the population subgroups under consideration, differences in such scores [40] were computed. When calculating these differences, the pooled standard deviation was obtained by weighting the specific sample size of the different groups involved in the comparisons [41]. The magnitudes of the resulting effect sizes were interpreted as trivial (*d* = 0.00 to 0.20), small (*d* = 0.20 to 0.50), intermediate (*d* = 0.50 to 0.80), or large (*d* > 0.80) [40].

Thirdly, evidence of convergent validity was obtained by computing zero-order correlations (*r*) between the BEECOM scores and scores on (i) physical appearance comparisons and (ii) three different ED outcomes (i.e., overall ED symptoms, weight and shape concerns, and dietary restraint). The magnitudes of the resulting effect sizes were interpreted as trivial (0.00 to 0.10), small (0.10 to 0.30), moderate (0.30 to 0.50), or large (>0.50) [40]. Fourthly, evidence of incremental validity was obtained by conducting a set of linear regression analysis in which (i) the scores derived from both versions of the BEECOM and (ii) BMI and PACS-R scores (as covariates) were introduced as independent variables, whereas three different ED outcomes (i.e., overall ED symptoms, weight and shape concerns, and dietary restraint) were introduced as dependent variables. Finally, possible differences in the strength of the multivariate relationships between the BEECOM scores and the ED outcomes across populations subgroups were examining using Model 1 of PROCESS macro for SPPS following the procedure described elsewhere [42]. This meant introducing two new groups of independent variables into the regression models described in this paragraph. The first such group consisted of a multi-categorical moderating variable involving the four study population subgroups. This variable was indicator-coded in PROCESS with the younger female group as the reference group, yielding three dummy variables (younger males vs. younger females [D_1_], older females vs. younger females [D_2_], and older males vs. younger females [D_3_]). The second of these groups consisted of the product terms between the predictor variable and the moderator (i.e., BEECOM scores x D_1–3_). This procedure tested the interactions involving each of the candidate predictors (i.e., each of the specific BEECOM scores) in separate models. As a result of this procedure, conditional standardized effects of BEECOM scores on the three ED outcomes under consideration (i.e., simple slopes) for each of the four population subgroups were obtained. In order to deal with eventual violations of some of the assumptions inherent in the application of regression techniques (e.g., homoscedasticity of variance or residual normality), the last two sets of analyses described in this paragraph were conducted by applying a bias-corrected and accelerated bootstrapping technique of 5000 resamples [22]. Missing data (0.72%) were handled using full information imputation methods implemented in Mplus version 7 (http://www.statmodel.com/index.shtml, accessed on 21 December 2022). A significance level of 0.05 was used in all the statistical analyses.

## 3. Results

### 3.1. Factorial Structure

**Figure 1:**
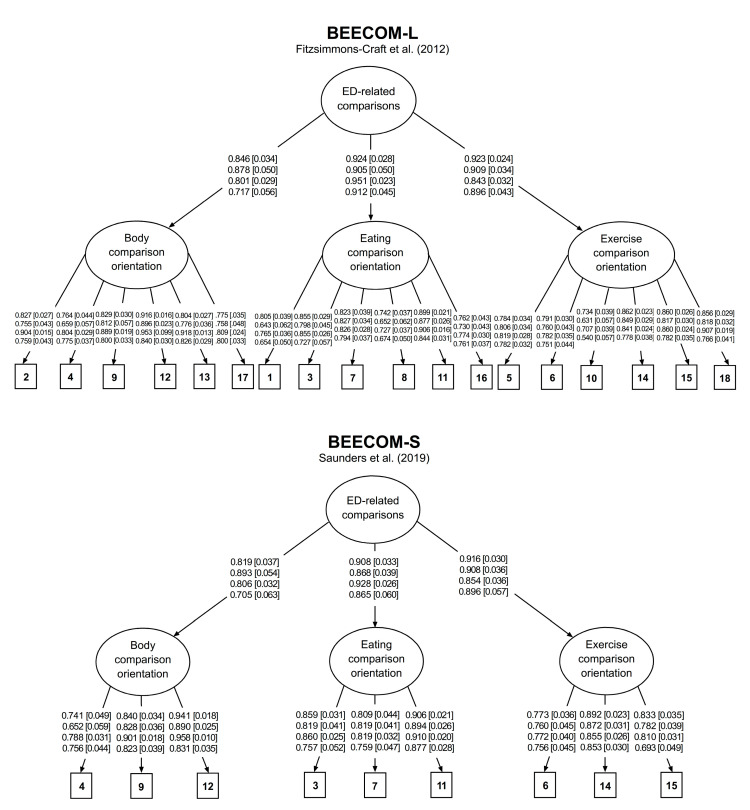
Factor structure for the long (BEECOM-L) and the short (BEECOM-S) versions of the Body, Eating, and Exercise Comparison Orientation Measure [16,17]. ED = eating disorders. The four rows of values correspond to the groups consisting of younger females, younger males, older females, and older males, respectively. Values outside square brackets represent standardized factor loadings (λ). Values inside square brackets represent standardized errors (SE).

Marginally acceptable and adequate goodness-of-fit indices were obtained for the BEECOM-L and the BEECOM-S, respectively, in all the population subgroups under consideration (see Table 2). Further inspection of MI derived from the CFA involving the BEECOM-L revealed the following items as the main sources of poor model fit: (i) Item 17 (body comparison orientation factor), which was found to cross-load on the exercise comparison orientation factor both in the older male (MI = 12.207) and in the younger (MI = 20.903) and older (MI = 24.780) female subsamples; (ii) Items 8 and 16 (eating comparison orientation factor), which were found to cross-load on the exercise comparison orientation factor in the younger male subsample (MI = 25.405) and in the older female subsample (MI = 15.192), respectively; and (iii) Item 10 (exercise comparison orientation factor), which was found to cross-load on the eating comparison orientation factor both in the younger male (MI = 15.885) and in the older female (MI = 13.135) subsamples. Standardized factor loadings for both versions of the BEECOM are shown in Figure 1. These ranged from 0.540 to 0.953 (first-order factors) and from 0.717 to 0.951 (higher-order factor) for the BEECOM-L, and from 0.652 to 958 (first-order factors) and from 0.705 to 0.928 (higher-order factor) for the BEECOM-S.

### 3.2. Invariance Analyses and Differences in BEECOM Scores across Population Subgroups

The results of invariance analysis (see Table 2) revealed substantial differences between the models under comparison only in those aimed at testing the invariant nature of items’ intercepts. These differences were no longer remarkable after removing the equality restriction for 8 of the 72 (BEECOM-L) and 4 of the 36 (BEECOM-S) intercepts under examination. The effect sizes of differences in study variables across population subgroups are shown in Table 3. In terms of ED-related social comparisons, mainly small-sized differences were found, which tended to favor the groups consisting of female and older participants. Of all scores derived from the BEECOM, differences of at least intermediate magnitude were observed only between older female and younger male groups in terms of body comparison orientation, which favored the former. Small differences were also found for several ED outcomes, which tended to favor the female groups.

### 3.3. Convergent Validity

The results of correlational analyses are shown in Table 4. Slightly lower inter-factor correlations were found for BEECOM-S compared to BEECOM-L, ranging from 0.022 (body orientation and eating orientation factors in the older female subsample) to 0.090 (eating orientation and exercise orientation in the older male subsample). Correlations between higher-order factors of both versions of the BEECOM ranged from 0.977 (younger male subsample) and 0.988 (older female subsample). Correlations between corresponding factors from the long and short versions of the BEECOM ranged from 0.943 (eating comparison orientation in the older male subsample) to 0.979 (body comparison orientation in the older female subsample). The scores from both versions of the BEECOM were positively correlated with the scores from both the PACS-R (which ranged from 0.416 to 0.787) and the different ED outcomes (which ranged from 0.242 to 0.713) and were largely consistent with the expected range of effect sizes.

### 3.4. Regression Analyses

#### 3.4.1. Incremental Validity

Results from the regression analyses aimed at providing evidence on incremental validity for the Spanish versions of the BEECOM-L and BEECOM-S are shown in Table 5. Minimum tolerance values were 0.369 (overall ED symptoms), 0.315 (weight and shape concerns), and 0.369 (dietary restraint), whereas maximum variance inflation factors (VIF) values 2.721 (overall ED symptoms), 3.171 (weight and shape concerns), and 2.721 (dietary restraint), indicating that multicollinearity was not an issue [43]. Both the total and subscale scores of the BEECOM accounted for additional variance above and beyond BMI and another comparison measure (i.e., the PACS-R) for the three ED outcomes under consideration. Inspection of the regression coefficients revealed that body comparison orientation was the subscale accounting for the largest amount of unique variance both in overall ED symptoms and weight and shape concerns, with the same being true for the eating comparison orientation subscale in the case of symptoms involving dietary restraint. In contrast, the exercise comparison orientation subscale accounted for the least amount of unique variance in all the three ED outcomes under consideration. The variance explained tended to be slightly higher in regression models using BEECOM-L scores than in those using BEECOM-S scores. The differences in explained variance (*R*^2^) between the models using scores derived from either version of the BEECOM ranged from 0.010 to 0.014 (total scores) and from 0.005 to 0.010 (subscale scores).

#### 3.4.2. Conditional Effects of the BEECOM Scores on ED Outcomes across Population Subgroups

The results of the moderation analyses examining the conditional effects of the BEECOM scores on ED outcomes across population subgroups are shown in Supplementary Materials (Tables S1–S3). *R*^2^ increases as a result of the interaction were statistically significant in three cases. Firstly, in models considering overall ED symptoms as the dependent variable that tested interactions involving total scores (∆*R*^2^ = 0.005, *F* [3, 1203], *p* = 0.046) or eating comparison orientation scores (∆*R*^2^ = 0.006, *F* [3, 1201], *p* = 0.043) derived from BEECOM-L. Secondly, in models considering weight and shape concern as the dependent variable that tested interactions involving (i) total scores derived both from the BEECOM-L (∆*R*^2^ = 0.007, *F* [3, 1203] = 3.840, *p* = 0.009) and the BEECOM-S (∆*R*^2^ = 0.007, *F* [3, 1203] = 3.496, *p* = 0.015); (ii) body comparison orientation scores derived both from the BEECOM-L (∆*R*^2^ = 0.006, *F* [3, 1201] = 3.763, *p* = 0.010) and the BEECOM-S (∆*R*^2^ = 0.005, *F* [3, 1200] = 2.909, *p* = 0.034); (iii) eating comparison orientation scores derived both from the BEECOM-L (∆*R*^2^ = 0.007, *F* [3, 1201] = 3.000, *p* = 0.009) and the BEECOM-S (∆*R*^2^ = 0.006, *F* [3, 1200] = 2.855, *p* = 0.036); and (iv) exercise comparison orientation derived from the BEECOM-S (∆*R*^2^ = 0.004, *F* [3, 1200] = 2.704, *p* = 0.044). Thirdly, in models considering dietary restraint as the dependent variable that tested the interaction involving body orientation comparison scores derived from the BEECOM-S (∆*R*^2^ = 0.006, *F* [3, 1201] = 2.734, *p* = 0.042).

The conditional effects of the subscale scores of both versions of the BEECOM on the ED outcomes across population subgroups are shown in Table 6. Largely similar patterns of positive contribution to explained variance were found for body and eating comparison orientation (for overall ED symptoms), body comparison orientation (for weight and shape concerns), and eating comparison orientation (for dietary restraint). However, these effects differed slightly across population subgroups in three cases: (i) for body comparison orientation, which—apart from tending to explain greater amount of variance in weight and shape concerns in younger groups—accounted for unique variance in dietary restraint only in the younger female group; (ii) eating comparison orientation, which—apart from tending to explain greater amount of variance in overall ED symptoms in younger groups—accounted for unique variance in weight and shape concerns in all groups except for the older male one; and (iii) and exercise comparison orientation, which accounted for unique variance in overall ED symptoms only in the younger female group.

## 4. Discussion

The present study elaborates on previous research on the psychometric of the BEECOM [16,17,18] by examining this is issue in both the long and short versions of the instrument in four different samples in terms of gender (i.e., male and female) and age (i.e., adolescents and young adults). The results supported the use of the BEECOM-S over BEECOM-L in the four population subgroups under consideration. This recommendation is based on the fact that although we found evidence that supports both versions in terms of reliability, convergent/incremental validity, and measurement invariance of their scores across gender and age groups, this same evidence clearly emerged in terms of factorial validity only in the case of the BEECOM-S. These findings are largely consistent with those reported for clinical and non-clinical samples in terms of ED consisting of females in their young adulthood [16] in suggesting the improved performance in terms of factorial validity of the BEECOM-S over the BEECOM-L. These findings make it possible to extend previous recommendations for the use of the BEECOM-S in clinical and non-clinical females in their young adulthood to other relevant populations in terms of ED prevention efforts, such as adolescents of both genders and young adult males not clinically diagnosed with ED [4,19]. The main findings of the present study are further elaborated below.

A first notable finding of the present study concerns the apparent mismatch of some of the items included in the instrument with their theoretical ascription factors. This was evident from the fact that the model mis-specification of the BEECOM-L was largely due to the ambiguous factor loadings shown by most of the items not included in the BEECOM-S. A possible explanation for these findings could be drawn by examining the content of this group of items. For example, the three items showing unexpected cross-loadings on the exercise comparison orientation factor include content that, despite alluding to comparison features from the original factors (i.e., the body or eating habits), could reflect a presumably healthy lifestyle focused on the attainment of a certain body ideal throughout dietary and exercise behavior [44,45]. This was the case with Item 17 (which alludes to having a toned body) and Items 8 and 16 (which allude to healthy eating and consuming junk food, respectively). The latter suggests that part of the content included in the items present in the BEECOM-L but excluded from the BEECOM-S may cover comparisons referred to more than one of the theoretical objects of comparison (e.g., food and exercise or body and exercise). In this vein, the similar correlation patterns observed between the scores derived from (i) both versions of the BEECOM and (ii) the variables included in the convergent validity analyses would suggest that the omission of items present only in the BEECOM-L does not undermine the comprehensiveness of the BEECOM-S scores. This means that despite some items being removed (these including the items potentially referred to more than one comparison feature), the key aspects of the constructs being assessed would not be missing, which would support the use of the reduced version of the instrument. Further studies should nevertheless examine whether complex comparisons incorporating elements of more than one of the objects of comparison under examination would be relevant within the context of studying the onset and maintenance of ED.

A second main implication from the present study derives from the evidence that supported the measurement invariance of the BEECOM-S scores across males/females and adolescents/young adults. This finding confirms with empirical data the applicability of the instrument to male individuals hypothesized by the authors who developed the original version of the BEECOM [17]. This also implies that the instrument can be recommended for the purpose of conducting reasonably unbiased comparisons between the population groups under consideration in terms of self-reported levels of body, eating, and exercise comparison orientation. In this vein, the pattern of differences observed in body-related comparisons between the age and gender groups considered in the present study is consistent with prior research [10,12,24]. We refer to the fact that body-related comparison scores favored females and individuals in their young adulthood over males and adolescents, respectively. In this sense, the results of the present study suggest that, while showing appreciably smaller differences, such a pattern could be equally present in comparisons concerning eating and exercise. Taken together, these findings suggest that females in their young adulthood may be a priority target for prevention efforts aimed at reducing body-focused social comparisons and, as a result, their potentially harmful outcomes [15,23,46].

A third important implication from the present study is that the three forms of social comparison assessed by the BEECOM may differ in their contribution to explaining specific ED outcomes across gender- and age-based population subgroups. In this vein, the results broadly suggest that comparisons focused on body, eating, and exercise may be particularly detrimental in terms of their potential influence on different ED outcomes in female individuals and, among these, in those in their adolescence. Consequently, this latter population emerges as a clear target for prevention in terms of the three forms of comparison under consideration. In this case, this is not due to the higher comparison frequency of comparison observed in these groups (as was found when analyzing the differences in the BEECOM scores between groups) but because of the positive and particularly strong relationship between this variable and the ED outcomes found in these groups. These results suggest call for further research on the role of gender and age in the relationship between the different forms of social comparisons proposed as particularly relevant in the context of ED and the symptoms inherent to this group of pathologies. The results derived from such investigations could inform both the content (e.g., by identifying priority comparison features) and the target population (e.g., in terms of age groups and gender) of ED prevention efforts and intervention development.

Several limitations of the present study should be acknowledged. Firstly, the data came only from the Spanish version of the BEECOM, which prevents the results obtained here from being generalized to all language versions of the instrument (i.e., the original English version [17] or the Farsi translation [18]). This is a relevant limitation given the lack of cross-country and cross-cultural equivalence shown by a number of instruments assessing body image-related constructs [47,48]. In the same vein, the fact that the study population was limited to adolescents and young adults does not allow the results obtained to be generalized to other populations of interest in terms of ED prevalence (e.g., middle-aged and older women [49]). Secondly, evidence suggesting the limited contribution of general comparison tendencies relative to physical appearance social comparisons in explaining variability in ED outcomes over and above the scores from the BEECOM subscales [17] led us to omit the former from the incremental validity analyses. However, it cannot be ruled out that the inclusion of a score reflecting general comparison trends would have led to slightly different results. A third and final limitation is the lack of testing of a relevant psychometric property such as reliability in terms of temporal stability. As the evidence on this issue is so far limited to that reported for college women in the case of the original English language version of the BEECOM-L [17], future research aimed at examining the reliability of the BEECOM-S in terms of temporal stability is warranted.

## 5. Conclusions

The BEECOM-S proves to be a psychometrically sound instrument for assessing body-, food- and exercise-related comparisons in non-clinical young individuals from Spain. These three types of comparisons could be important targets within the context of ED prevention in adolescent and young adult males and, particularly, females.

## Figures and Tables

**Table 1:** Composite reliability (ρ) and internal consistency (*α*) of study measures across population subgroups.

			Younger Females (*n* = 287)		Older Females (*n* = 360)		Younger Males (*n* = 295)		Older Males (*n* = 271)	
Instrument	Dimensionality	Factor	ρ	α	ρ	α	ρ	α	ρ	α
BEECOM-L	Higher-Order	ED-related social comparison	0.926	0.963	0.901	0.963	0.925	0.956	0.882	0.940
BEECOM-L	First-Order (Factor 1)	Body comparison orientation	0.928	0.928	0.954	0.954	0.902	0.905	0.914	0.915
BEECOM-L	First-Order (Factor 2)	Eating comparison orientation	0.923	0.921	0.920	0.917	0.890	0.887	0.881	0.876
BEECOM-L	First-Order (Factor 3)	Exercise comparison orientation	0.923	0.922	0.925	0.926	0.904	0.900	0.876	0.875
BEECOM-S	Higher-Order	ED-related social comparison	0.913	0.917	0.898	0.921	0.919	0.906	0.865	0.882
BEECOM-S	First-Order (Factor 1)	Body comparison orientation	0.881	0.876	0.915	0.910	0.837	0.825	0.846	0.845
BEECOM-S	First-Order (Factor 2)	Eating comparison orientation	0.894	0.892	0.898	0.895	0.882	0.879	0.841	0.832
BEECOM-S	First-Order (Factor 3)	Exercise comparison orientation	0.872	0.869	0.854	0.854	0.847	0.847	0.813	0.807
PACS-R	Unidimensional	Physical appearance comparisons	0.965	0.953	0.980	0.969	0.962	0.943	0.972	0.958
EDE-QS	Unidimensional	Overall ED symptoms	0.941	0.890	0.941	0.889	0.936	0.865	0.932	0.864
EDE-QS	Sub-scale (items 4, 5 & 6)	Weight and shape concerns	0.803	0.790	0.793	0.807	0.743	0.707	0.703	0.693
EDE-QS	Sub-scale (items 1 & 2)	Dietary restraint	0.777	0.622	0.642	0.508	0.657	0.478	0.632	0.469

Note. PACS-R = Physical Appearance Comparison Scale-Revised; BEECOM = Body, Eating, and Exercise Comparison Orientation Measure (-L = Long version, -S = Short version); EDE-QS = Eating Disorder Examination Questionnaire Short; ED = eating disorders.

**Table 2:** Goodness-of-fit indexes across population subgroups and invariance testing of the long and short versions of the BEECOM.

Version	Population/Model	χ^2^	*df*	χ^2^/*df*	Comparison Models	CFI	ΔCFI	RMSEA				SRMR
								Est.	90% CI		*p*	
									Lower	Upper		
Long	Younger females (*n* = 287)	381.252	132	2.888	-	0.934	-	0.072	0.064	0.081	0.000	0.051
	Older females (*n* = 360)	293.203	132	2.221	-	0.934	-	0.065	0.055	0.075	0.007	0.049
	Younger males (*n* = 295)	305.732	132	2.316	-	0.910	-	0.070	0.059	0.080	0.001	0.052
	Older males (*n* = 271)	238.592	132	1.808	-	0.941	-	0.052	0.042	0.063	0.348	0.056
	M0: Baseline model (without invariance)	1205.833	528	2.284	-	0.931	-	0.065	0.060	0.070	0.000	0.052
	M1: Invariant first-order factor loadings	1273.711	573	2.223	M1 vs. M0	0.929	−0.002	0.064	0.059	0.068	0.000	0.057
	M2: M1 + Invariant higher-order factor loadings	1290.103	579	2.228	M2 vs. M1	0.928	−0.001	0.064	0.059	0.068	0.000	0.061
	M3: M2 + Invariant items’ intercepts	1516.660	630	2.407	M3 vs. M2	0.910	−0.028 *	0.068	0.064	0.073	0.000	0.066
	M3P: M2 + Partially invariant items’ intercepts ^a^	1427.879	622	2.296	M3P vs. M2	0.918	−0.010	0.065	0.061	0.070	0.000	0.064
	M4: M3P + Invariant first-order factor intercepts	1454.815	625	2.328	M4 vs. M3P	0.916	−0.002	0.066	0.062	0.071	0.000	0.078
	M5: M4 + Invariant first-order factor disturbances	1475.005	634	2.327	M5 vs. M4	0.914	−0.002	0.066	0.062	0.071	0.000	0.078
	M6: M5 + Invariant item residual variances	1544.027	688	2.244	M6 vs. M5	0.913	−0.001	0.064	0.060	0.068	0.000	0.082
	Younger females (*n* = 287)	47.988	24	2.000	-	0.983	-	0.053	0.030	0.074	0.390	0.032
	Older females (*n* = 360)	40.702	24	1.696	-	0.983	-	0.049	0.020	0.075	0.487	0.035
	Younger males (*n* = 295)	30.235	24	1.260	-	0.991	-	0.031	0.000	0.061	0.827	0.029
	Older males (*n* = 271)	28.854	24	1.202	-	0.993	-	0.026	0.000	0.056	0.890	0.033
Short	M0: Baseline model (without invariance)	145.661	96	1.517	-	0.987	-	0.041	0.027	0.054	0.855	0.032
	M1: Invariant first-order factor loadings	161.685	114	1.418	M1 vs. M0	0.987	−0.000	0.037	0.023	0.050	0.954	0.036
	M2: M1 + Invariant higher-order factor loadings	173.387	120	1.445	M2 vs. M1	0.986	0.001	0.038	0.025	0.050	0.944	0.044
	M3: M2 + Invariant items’ intercepts	274.654	144	1.907	M3 vs. M2	0.965	−0.021 *	0.055	0.045	0.064	0.209	0.056
	M3P: M2 + Partially invariant items’ intercepts ^b^	230.563	140	1.647	M3P vs. M2	0.976	−0.010	0.046	0.035	0.057	0.714	0.047
	M4: M3P + Invariant first-order factor intercepts	247.490	143	1.731	M4 vs. M3P	0.972	−0.004	0.049	0.039	0.059	0.546	0.068
	M5: M4 + Invariant first-order factor disturbances	261.440	152	1.720	M5 vs. M4	0.971	−0.001	0.049	0.039	0.059	0.571	0.068
	M6: M5 + Invariant item residual variances	277.779	179	1.552	M6 vs. M5	0.974	0.003	0.043	0.032	0.052	0.894	0.071

Note. CFI = comparative fit index; RMSEA = root mean square error of approximation; CI = confidence interval; *df* = degrees of freedom; Est. = estimate; Lo = lower; up = Upper; SRMR = standardized root mean square residual; M = model. * ∆CFI < −0.01 (signals lack of invariance). ^a^ Intercepts of items 5, 11, 15, and 18 (younger males), Item 13 (older females) and Items 6, 15, and 18 (older males) are freed. ^b^ Intercepts of Items 9 and 12 (younger males) and Items 6 and 15 (older males) are freed.

**Table 3:** Scores of Study Variables and Effect Sizes of Differences Across Population Subgroups.

		Younger Females (*n* = 287)		Older Females (*n* = 360)		Younger Males (*n* = 295)		Older Males (*n* = 271)		Younger vs. Older Females	Younger Females vs. Younger Males	Younger Females vs. Older Males	Older Females vs. Younger Males	Older Females vs. Older Males	Younger vs. Older Males
	Range ^a^	*M*	*SD*	*M*	*SD*	*M*	*SD*	*M*	*SD*	*d*	*d*	*d*	*d*	*d*	*d*
Percentile-BMI	0–99.40	50.353	27.862	50.464	27.564	58.405	27.530	54.597	26.811	0.004	0.290	0.155	0.288	0.152	−0.140
BEECOM-L (Total score)	1–7	2.463	1.426	2.888	1.482	2.240	1.263	2.544	1.131	0.292	−0.166	0.063	−0.467	−0.256	0.253
BEECOM-L (Body)	1–7	2.859	1.702	3.345	1.809	2.306	1.392	2.574	1.340	0.276	−0.356	−0.186	−0.636	−0.475	0.196
BEECOM-L (Eating)	1–7	2.230	1.490	2.738	1.590	2.054	1.300	2.437	1.282	0.328	−0.126	0.149	−0.466	−0.205	0.297
BEECOM-L (Exercise)	1–7	2.300	1.476	2.580	1.524	2.366	1.460	2.621	1.278	0.186	0.045	0.233	−0.143	0.029	0.185
BEECOM-S (Total score)	1–7	2.509	1.478	2.924	1.529	2.302	1.362	2.623	1.194	0.275	−0.146	0.084	−0.427	−0.215	0.250
BEECOM-S (Body)	1–7	2.947	1.777	3.403	1.850	2.410	1.530	2.654	1.433	0.251	−0.324	−0.181	−0.579	−0.445	0.164
BEECOM-S (Eating)	1–7	2.152	1.566	2.620	1.696	2.036	1.454	2.356	1.377	0.285	−0.077	0.138	−0.367	−0.168	0.225
BEECOM-S (Exercise)	1–7	2.427	1.622	2.748	1.629	2.449	1.598	2.857	1.437	0.197	0.014	0.280	−0.185	0.070	0.268
PACS-R	0–4	1.162	0.991	1.356	1.104	0.967	0.888	1.062	0.916	0.184	−0.207	−0.105	−0.384	−0.286	0.105
Overall ED Symptoms	0–3	0.685	0.624	0.651	0.590	0.550	0.567	0.536	0.505	−0.056	−0.226	−0.261	−0.174	−0.207	−0.026
Weight and shape concern	0–3	0.880	0.912	0.651	0.590	0.587	0.746	0.581	0.691	−0.012	−0.352	−0.368	−0.341	−0.110	−0.008
Dietary Restraint	0–3	0.643	0.810	0.658	0.743	0.539	0.729	0.548	0.673	0.019	−0.135	−0.127	−0.161	−0.154	0.013

Note. BMI = body mass index; BEECOM = Body, Eating, and Exercise Comparison Orientation Measure (-L = Long version, -S = Short version); PACS-R = Physical Appearance Comparison Scale-Revised; ED = eating disorders. ^a^ Observed range in the case of Percentile-BMI and possible range for the remaining variables.

**Table 4:** Results of Correlational Analyses.

	1	2	3	4	5	6	7	8	9	10	11	12	13
1. Percentile-BMI	-	0.000 0.080	0.078 0.053	−0.029 0.046	−0.048 0.111	0.032 0.086	0.040 0.059	−0.053 0.036	−0.056 0.121 ^a^	−0.066 0.042	0.219 ^c^ 0.297 ^c^	0.196 ^b^ 0.331 ^c^	0.299 ^c^ 0.305 ^c^
2. BEECOM-L (Total score)	0.147 ^a^ 0.240	-	0.905 ^c^ 0.837	0.913 ^c^ 0.889 ^c^	0.920 ^c^ 0.886 ^c^	0.977 ^c^ 0.982 ^c^	0.862 ^c^ 0.789 ^c^	0.853 ^c^ 0.844 ^c^	0.880 ^c^ 0.851 ^c^	0.691 ^c^ 0.601 ^c^	0.584 ^c^ 0.520 ^c^	0.555 ^c^ 0.398 ^c^	0.325 ^c^ 0.337 ^c^
3. BEECOM-L (Body)	0.146 ^a^ 0.283	0.911 ^c^ 0.899 ^c^	-	0.734 ^c^ 0.589 ^c^	0.734 ^c^ 0.583 ^c^	0.883 ^c^ 0.835 ^c^	0.953 ^c^ 0.966 ^c^	0.696 ^c^ 0.587 ^c^	0.694 ^c^ 0.557 ^c^	0.691 ^c^ 0.710 ^c^	0.567 ^c^ 0.514 ^c^	0.577 ^c^ 0.430 ^c^	0.278 ^c^ 0.288 ^c^
4. BEECOM-L (Eating)	0.113 0.180 ^b^	0.920 ^c^ 918 ^c^	0.739 ^c^ 0.732 ^c^	-	0.772 ^c^ 0.738 ^c^	0.898 ^c^ 0.863 ^c^	0.710 ^c^ 0.529 ^c^	0.947 ^c^ 0.943 ^c^	0.741 ^c^ 0.719 ^c^	0.570 ^c^ 0.416 ^c^	0.565 ^c^ 0.415 ^c^	0.515 ^c^ 0.271 ^c^	0.343 ^c^ 0.270 ^c^
5. BEECOM-L (Exercise)	0.145 ^a^ 0.175 ^b^	0.920 ^c^ 0.890 ^c^	0.740 ^c^ 0.672 ^c^	0.806 ^c^ 0.765 ^c^	-	0.894 ^c^ 0.865 ^c^	0.696 ^c^ 0.553 ^c^	0.702 ^c^ 0.679 ^c^	0.961 ^c^ 0.953 ^c^	0.626 ^c^ 0.434 ^c^	0.473 ^c^ 0.426 ^c^	0.432 ^c^ 0.332 ^c^	0.273 ^c^ 0.324 ^c^
6. BEECOM-S (General)	0.136 ^a^ 0.246 ^c^	0.984 ^c^ 0.988 ^c^	0.910 ^c^ 0.897 ^c^	0.897 ^c^ 0.904 ^c^	0.899 ^c^ 0.873 ^c^	-	0.884 ^c^ 0.815 ^c^	0.883 ^c^ 0.857 ^c^	0.890 ^c^ 0.860 ^c^	0.690 ^c^ 0.610 ^c^	0.551 ^c^ 0.517 ^c^	0.510 ^c^ 0.404 ^c^	0.312 ^c^ 0.335 ^c^
7. BEECOM-S (Body)	0.137 ^a^ 0.290 ^c^	0.852 ^c^ 0.870 ^c^	0.966 ^c^ 0.979 ^c^	0.673 ^c^ 0.703 ^c^	0.677 ^c^ 0.642 ^c^	0.886 ^c^ 0.889 ^c^	-	0.677 ^c^ 0.531 ^c^	0.668 ^c^ 0.525 ^c^	0.657 ^c^ 0.682 ^c^	0.545 ^c^ 0.491 ^c^	0.533 ^c^ 0.424 ^c^	0.267 ^c^ 0.268 ^c^
8. BEECOM-S (Eating)	0.101 0.187 ^c^	0.894 ^c^ 0.891 ^c^	0.734 ^c^ 0.729 ^c^	0.965 ^c^ 0.967 ^c^	0.771 ^c^ 0.725 ^c^	0.900 ^c^ 0.904 ^c^	0.685 ^c^ 0.710 ^c^	-	0.677 ^c^ 0.648 ^c^	0.569 ^c^ 0.429 ^c^	0.512 ^c^ 0.433 ^c^	0.449 ^c^ 0.302 ^c^	0.318 ^c^ 0.273 ^c^
9. BEECOM-S (Exercise)	0.124 ^a^ 0.169 ^b^	0.894 ^c^ 0.866 ^c^	0.721 ^c^ 0.657 ^c^	0.783 ^c^ 0.739 ^c^	0.971 ^c^ 0.974 ^c^	0.895 ^c^ 0.867 ^c^	0.665 ^c^ 0.629 ^c^	0.743 ^c^ 0.698 ^c^	-	0.612 ^c^ 0.429 ^c^	0.405 ^c^ 0.384 ^c^	0.369 ^c^ 0.295 ^c^	0.242 ^c^ 0.306 ^c^
10. PACS-R	0.159 ^b^ 0.291 ^b^	0.776 ^c^ 0.746 ^c^	0.773 ^c^ 0.772 ^c^	0.668 ^c^ 0.635 ^c^	0.683 ^c^ 0.598 ^c^	0.787 ^c^ 738 ^c^	0.763 ^c^ 0.759 ^c^	0.689 ^c^ 0.635 ^c^	0.650 ^c^ 0.557 ^c^	-	0.326 ^c^ 0.354 ^c^	0.314 ^c^ 0.279 ^c^	0.113 0.184 ^b^
11. Overall ED Symptoms	0.279 ^c^ 0.387 ^c^	0.713 ^c^ 0.629 ^c^	0.673 ^c^ 0.605 ^c^	0.645 ^c^ 0.572 ^c^	0.641 ^c^ 0.518 ^c^	0.697 ^c^ 0.624 ^c^	0.634 ^c^ 0.599 ^c^	0.641 ^c^ 0.561 ^c^	0.592 ^c^ 0.493 ^c^	0.615 ^c^ 0.579 ^c^	-	0.880 ^c^ 0.873 ^c^	0.728 ^c^ 0.763 ^c^
12. Weight/Shape Concern	0.314 ^c^ 0.427 ^c^	0.636 ^c^ 0.573 ^c^	0.631 ^c^ 0.543 ^c^	0.554 ^c^ 0.538 ^c^	0.557 ^c^ 0.465 ^c^	0.626 ^c^ 0.565 ^c^	0.603 ^c^ 0.538 ^c^	0.559 ^c^ 0.523 ^c^	0.513 ^c^ 0.436 ^c^	0.563 ^c^ 0.546 ^c^	0.895 ^c^ 0.910 ^c^	-	0.542 ^c^ 0.636 ^c^
13. Dietary Restraint	0.372 ^c^ 0.370 ^c^	0.545 0.467 ^c^	0.484 ^c^ 0.404 ^c^	0.518 ^c^ 0.463 ^c^	0.500 ^c^ 0.400 ^c^	0.518 ^c^ 0.469 ^c^	0.436 ^c^ 0.402 ^c^	0.505 ^c^ 0.465 ^c^	0.452 ^c^ 0.382 ^c^	0.489 ^c^ 0.395 ^c^	0.817 ^c^ 0.773 ^c^	0.702 ^c^ 0.661 ^c^	-

Note. BMI = body mass index; PACS-R = Physical Appearance Comparison Scale-Revised; BEECOM = Body, Eating, and Exercise Comparison Orientation Measure (-L = Long version, -S = Short version); ED = eating disorders. Values for female (male) subsamples are presented below (above) the diagonal. The first (second) value in the cell corresponds to the younger (older) subsample. ^a^ *p* < 0.05; ^b^ *p* < 0.01; ^c^ *p* < 0.001.

**Table 5:** Results of regression analyses predicting eating disorder outcomes.

Independent Variables	Overall ED Symptoms							Weight and Shape Concerns							Dietary Restraint						
	*B (SE)*	*β*	*t*	*p*	LLCI	ULCI	*R^2^*	*B (SE)*	*β*	*t*	*p*	LLCI	ULCI	*R^2^*	*B (SE)*	*β*	*t*	*p*	LLCI	ULCI	*R^2^*
BEECOM-L (Higher order factor)							0.433							0.375							0.269
Constant	−0.265 (0.032)	-	−7.671	<0.001	−0.326	−0.326		−0.444 (0.047)	−	−8.577	<0.001	−0.534	−0.346		−0.365 (0.043)	−	−7.238	<0.001	−0.450	−0.283	
Percentile-BMI	0.005 (0.000)	0.216	9.901	<0.001	0.004	0.004		0.007 (0.001)	0.235	10.289	<0.001	0.006	0.008		0.008 (0.001)	0.283	11.440	<0.001	0.006	0.009	
PACS-R	0.050 (0.021)	0.086	2.752	0.014	0.009	0.091		0.093 (0.030)	0.112	3.413	0.001	0.033	0.153		0.001 (0.028)	0.001	0.035	0.972	−0.054	0.057	
BEECOM-L (Total)	0.225 (0.016)	0.532	16.998	<0.001	0.195	0.255		0.275 (0.022)	0.453	13.811	<0.001	0.230	0.319		0.219 (0.022)	0.402	11.312	<0.001	0.178	0.259	
BEECOM-S (Higher order factors)							0.419							0.363							0.259
Constant	−0.241 (0.031)	-	−6.958	<0.001	−0.302	−0.181		−0.410 (0.047)	-	−7.894	<0.001	−0.501	−0.320		−0.343 (0.042)	-	−6.810	<0.001	−0.425	−260	
Percentile-BMI	0.005 (0.000)	0.220	9.990	<0.001	0.004	0.006		0.007 (0.001)	0.239	10.361	<0.001	0.006	0.009		0.008 (0.001)	0.287	11.521	<0.001	0.006	0.009	
PACS-R	0.061 (0.021)	0.105	3.320	0.005	0.020	0.102		0.111 (0.030)	0.133	4.022	<0.001	0.053	0.171		0.011 (0.029)	0.015	0.423	0.672	−0.048	0.068	
BEECOM-S (Total)	0.205 (0.015)	0.505	15.975	<0.001	0.176	0.233		0.245 (0.021)	0.424	12.784	<0.001	0.202	0.288		0.119 (0.020)	0.383	10.719	<0.001	0.161	0.239	
BEECOM-L (First order factors)							0.438							0.392							0.273
Constant	−0.267 (0.031)	−	−7.758	<0.001	−0.327	−0.203		−0.451 (0.048)	−	−8.800	<0.001	−0.546	−0.354		−0.358 (0.042)	-	−7.103	<0.001	−0.442	−0.275	
Percentile-BMI	0.004 (0.000)	0.214	9.862	<0.001	0.004	0.005		0.007 (0.001)	0.232	10.225	<0.001	0.005	0.008		0.008 (0.001)	0.288	11.609	<0.001	0.006	0.009	
PACS-R	0.031 (0.022)	0.053	1.610	0.161	−0.012	0.075		0.046 (0.032)	0.056	1.618	0.135	−0.020	0.112		0.017 (0.029)	0.023	0.610	0.542	−0.040	0.076	
BEECOM-L (Body)	0.113 (0.015)	0.320	8.344	<0.001	0.084	0.142		0.183 (0.023)	0.364	9.100	<0.001	0.138	0.227		0.042 (0.021)	0.093	2.134	0.033	0.002	0.082	
BEECOM-L (Eating)	0.086 (0.017)	0.215	5.789	<0.001	0.052	0.121		0.093 (0.024)	0.163	4.209	<0.001	0.046	0.139		0.128 (0.024)	0.250	5.905	<0.001	0.080	0.177	
BEECOM-L (Exercise)	0.033 (0.015)	0.082	2.323	0.035	0.002	0.062		0.013 (0.023)	0.023	0.0622	0.569	−0.030	0.057		0.042 (0.022)	0.082	2.055	0.040	0.000	0.085	
BEECOM-S (Higher order factors)							0.428							0.382							0.268
Constant	−0.238 (0.031)	-	−6.885	<0.001	−0.301	−0.177		−0.412 (0.048)	−	−8.001	<0.001	−0.506	−0.317		−324 (−043)	-	−6.441	<0.001	−0.409	−0.242	
Percentile-BMI	0.005 (0.000)	0.219	10.000	<0.001	0.004	0.006		0.007 (0.001)	0.236	10.342	<0.001	0.006	0.008		0.008 (0.001)	0.291	11.699	<0.001	0.007	0.009	
PACS-R	0.041 (0.022)	0.071	2.149	0.032	−0.001	0.084		0.067 (0.030)	0.081	2.349	0.019	0.006	0.126		0.023 (0.029)	0.030	0.812	0.438	−0.034	0.078	
BEECOM-S (Body)	0.102 (0.013)	0.302	8.449	<0.001	0.077	0.127		0.161 (0.020)	0.333	8.932	<0.001	0.121	0.199		0.037 (0.018)	0.085	2.104	0.042	0.002	0.072	
BEECOM-S (Eating)	0.092 (0.015)	0.249	7.363	<0.001	0.063	0.122		0.101 (0.021)	0.190	5.395	<0.001	0.061	0.142		0.126 (0.021)	0.263	6.868	<0.001	0.085	0.168	
BEECOM-S (Exercise)	0.018 (0.012)	0.050	1.564	0.118	−0.006	0.042		0.000 (0.019)	0.001	0.021	0.983	−0.036	0.037		0.022 (0.018)	0.070	1.954	0.070	−0.003	0.068	

Note. ED = eating disorders; B = unstandardized regression coefficients; *SE* = standard error; *β* = standardized regression coefficients; LLCI, lower limit 95% bootstrap confidence interval; ULCI, upper limit 95% bootstrap confidence interval; *R^2^* = explained variance; BEECOM = Body, Eating, and Exercise Comparison Orientation Measure (-L = long version, -S = short version); PACS-R = Physical Appearance Comparison Scale-Revised.

**Table 6:** Conditional Effects of BEECOM scores on eating disorder outcomes across population subgroups.

			BEECOM-L					BEECOM-S				
IV	DV	Subgroup	*β* (*SE*)	*t*	*p*	LLCI	ULCI	*β* (*SE*)	*t*	*p*	LLCI	ULCI
BEECOM: Total	Overall ED symptoms	Younger females	0.375 (0.032)	11.802	<0.001	0.313	0.437	0.362 (0.033)	11.049	<0.001	0.298	0.426
		Younger males	0.329 (0.035)	9.314	<0.001	0.259	0.398	0.297 (0.032)	8.793	<0.001	0.231	0.364
		Older females	0.280 (0.030)	9.195	<0.001	0.220	0.339	0.273 (0.030)	9.054	<0.001	0.214	0.332
		Older males	0.275 (0.041)	6.788	<0.001	0.196	0.355	0.263 (0.042)	6.228	<0.001	0.180	0.346
BEECOM: Body	Overall ED symptoms	Younger females	0.232 (0.034)	6.916	<0.001	0.166	0.298	0.212 (0.032)	6.541	<0.001	0.148	0.275
		Younger males	0.200 (0.043)	4.687	<0.001	0.116	0.284	0.174 (0.038)	4.589	<0.001	0.099	0.248
		Older females	0.140 (0.031)	4.467	<0.001	0.079	0.202	0.132 (0.030)	4.402	<0.001	0.073	0.190
		Older males	0.171 (0.039)	4.398	<0.001	0.094	0.247	0.153 (0.038)	4.051	<0.001	0.079	0.227
BEECOM: Eating	Overall ED symptoms	Younger females	0.191 (0.036)	5.272	<0.001	0.120	0.262	0.218 (0.036)	6.028	<0.001	0.147	0.289
		Younger males	0.181 (0.043)	4.184	<0.001	0.096	0.266	168 (0.038)	4.394	<0.001	0.093	0.242
		Older females	0.105 (0.031)	3.406	0.001	0.044	0.165	0.120 (0.030)	4.028	<0.001	0.062	0.179
		Older males	0.088 (0.038)	2.317	0.021	0.013	0.162	0.122 (0.039)	3.103	0.002	0.045	0.198
BEECOM: Exercise	Overall ED symptoms	Younger females	0.113 (0.036)	3.147	0.002	0.042	0.183	0.100 (0.032)	3.108	0.002	0.037	0.163
		Younger males	0.054 (0.033)	1.645	0.100	−0.010	0.119	0.014 (0.032)	0.441	0.659	−0.049	0.077
		Older females	0.019 (0.034)	0.577	0.564	−0.047	0.086	0.018 (0.030)	0.601	0.548	−0.041	0.078
		Older males	0.021 (0.036)	0.573	0.567	−0.050	0.092	0.005 (0.033)	0.157	0.875	−0.060	0.070
BEECOM: Total	Weight and shape concerns	Younger females	0.469 (0.045)	10.497	<0.001	0.381	0.556	0.455 (0.046)	9.873	<0.001	0.365	0.546
		Younger males	0.396 (0.047)	8.451	<0.001	0.304	0.488	0.347 (0.046)	7.513	<0.001	0.257	0.438
		Older females	0.355 (0.047)	8.248	<0.001	0.270	0.439	0.341 (0.043)	7.908	<0.001	0.256	0.426
		Older males	0.261 (0.055)	4.709	<0.001	0.152	0.369	0.252 (0.057)	4.397	<0.001	0.140	0.364
BEECOM: Body	Weight and shape concerns	Younger females	0.361 (0.049)	7.407	<0.001	0.265	0.456	0.331 (0.046)	7.173	<0.001	0.241	0.422
		Younger males	0.312 (0.062)	5.009	<0.001	0.190	0.435	0.252 (0.056)	4.512	<0.001	0.142	0.361
		Older females	0.213 (0.048)	4.449	<0.001	0.119	0.307	0.195 (0.045)	4.320	<0.001	0.107	0.284
		Older males	0.200 (0.057)	3.509	<0.001	0.088	0.312	0.182 (0.055)	3.331	0.001	0.075	0.290
BEECOM: Eating	Weight and shape concerns	Younger females	0.216 (0.052)	4.161	<0.001	0.114	0.317	0.263 (0.051)	5.126	<0.001	0.162	0.363
		Younger males	0.194 (0.054)	3.557	<0.001	0.087	0.301	0.172 (0.050)	3.460	0.001	0.074	0.269
		Older females	0.146 (0.045)	3.230	0.001	0.057	0.234	0.164 (0.044)	3.690	<0.001	0.077	0.251
		Older males	0.012 (0.052)	0.232	0.817	−0.090	0.114	0.069 (0.052)	1.334	0.182	−0.033	0.172
BEECOM: Exercise	Weight and shape concerns	Younger females	0.129 (0.050)	2.564	0.010	0.030	0.227	0.112 (0.045)	2.455	0.014	0.022	0.201
		Younger males	0.046 (0.045)	1.022	0.307	−0.043	0.135	−0.003 (0.044)	−0.065	0.948	−0.089	0.083
		Older females	0.026 (0.048)	00.544	0.587	−0.068	0.120	0.018 (0.043)	0.408	0.683	−0.067	0.103
		Older males	−0.030 (0.051)	−0.594	0.553	−0.130	0.069	−0.049 (0.047)	−1.044	0.297	−0.140	0.043
BEECOM: Total	Dietary restraint	Younger females	0.397 (0.047)	8.403	<0.001	0.304	0.490	0.375 (0.049)	7.693	<0.001	0.280	0.471
		Younger males	0.260 (0.046)	5.606	<0.001	0.169	0.351	0.245 (0.047)	5.231	<0.001	0.153	0.337
		Older females	0.277 (0.040)	6.868	<0.001	0.198	0.357	0.274 (0.041)	6.732	<0.001	0.194	0.354
		Older males	0.258 (0.055)	4.708	<0.001	0.151	0.366	0.247 (0.056)	4.429	<0.001	0.138	0.356
BEECOM: Body	Dietary restraint	Younger females	0.142 (0.048)	2.959	0.003	0.048	0.236	0.120 (0.048)	2.516	0.012	0.026	0.214
		Younger males	−0.005 (0.056)	−0.094	0.925	−0.116	0.105	−0.006 (0.051)	−0.126	0.900	−0.107	0.094
		Older females	0.018 (0.042)	0.424	0.672	−0.065	0.100	0.018 (0.041)	0.432	0.666	−0.062	0.097
		Older males	0.043 (0.059)	0.723	0.470	−0.074	0.160	0.036 (0.055)	0.650	0.516	−0.072	0.144
BEECOM: Eating	Dietary restraint	Younger females	0.282 (0.054)	5.258	<0.001	0.177	0.388	0.298 (0.053)	5.590	<0.001	0.193	0.402
		Younger males	0.182 (0.053)	3.446	0.001	0.078	0.285	0.181 (0.051)	3.524	<0.001	0.080	0.282
		Older females	0.181 (0.043)	4.186	<0.001	0.096	0.266	0.199 (0.042)	4.743	<0.001	0.117	0.281
		Older males	0.109 (0.057)	1.903	0.057	−0.003	0.221	0.131 (0.058)	2.243	0.025	0.016	0.245
BEECOM: Exercise	Dietary restraint	Younger females	0.168 (0.051)	3.275	0.001	0.067	0.269	0.143 (0.048)	2.979	0.003	0.049	0.237
		Younger males	0.041 (0.046)	0.892	0.372	−0.050	0.133	0.016 (0.046)	0.349	0.727	−0.074	0.106
		Older females	0.054 (0.045)	1.197	0.232	−0.034	0.142	0.050 (0.042)	1.202	0.230	−0.032	0.133
		Older males	0.049 (0.050)	0.988	0.323	−0.049	0.148	0.042 (0.052)	0.810	0.418	−0.060	0.145

Note. DV = dependent variable; IV = independent variable; *β* = standardized regression coefficients; *SE* = standard error; *R^2^* = explained variance; ED = eating disorders; BEECOM = Body, Eating, and Exercise Comparison Orientation Measure (-L = long version, -S = short version); LLCI = lower limit 95% bootstrap confidence interval; ULCI = upper limit 95% bootstrap confidence interval.

## Data Availability

The data presented in this study are available on request from the corresponding author.

## Supplementary Materials

The following supporting information can be downloaded at: https://www.mdpi.com/article/10.3390/nu15030626/s1, File S1: Spanish version of the BEECOM; Table S1: Results of moderation analyses examining the conditional effects of BEECOM scores on overall eating disorders symptoms; Table S2: Results of moderation analyses examining the conditional effects of BEECOM scores on weight and shape concerns; Table S3: Results of moderation analyses examining the conditional effects of BEECOM scores on dietary restraint.
