# Supplementary material for: Psychometric Examination of the Body, Eating, and Exercise Comparison Orientation Measure (BEECOM) among Spanish Adolescents and Young Adults
Source: Nutrients. 2023 Jan 26;15(3):626. doi: 10.3390/nu15030626 (PMC9921885; doi:10.3390/nu15030626)
Supplement: Supplementary file 1 [file nutrients-15-00626-s001.zip › File S1.pdf]

File S1

Psychometric examination of the Body, Eating, and Exercise Comparison Orientation Measure (BEECOM) among Spanish adolescents and young adults

Spanish version of the BEECOM

Por favor, puntúe cada uno de los siguientes aspectos relacionados con la frecuencia con la que se compara con sus compañeros/as del mismo sexo en términos de apariencia, ejercicio y alimentación. Recuerde que no hay respuestas correctas o incorrectas, así que responda de la forma más sincera posible.

En cuanto a los ítems que se refieren a las comparaciones que podrían hacerse al realizar ejercicio (por ejemplo, correr al aire libre, practicar un deporte organizado, o usar una máquina de cardio en un gimnasio): Si no está haciendo ejercicio en la actualidad, piense en las veces en las que ha hecho ejercicio (por ejemplo, ha participado en una clase de educación física, ha practicado un deporte organizado, ha caminado o corrido al aire libre) y responda en consecuencia.

	Nunca	Casi nunca	Rara vez	A veces	A menudo	Casi siempre	Siempre
1. Comparo la cantidad de comida que me dejo en el plato al terminar de comer con la que dejan los demás……………………………………………………....	1	2	3	4	5	6	7
2. Presto atención a si estoy más o menos delgado/a que el resto de la gente..…..	1	2	3	4	5	6	7
3. Durante las comidas, comparo lo que como con lo que comen otras personas.	1	2	3	4	5	6	7
4. En situaciones sociales, pienso en si mi cuerpo está a la altura del cuerpo de la gente……………………………………………………………………...…..	1	2	3	4	5	6	7
5. Cuando hago ejercicio, presto atención a cuánto tiempo hace ejercicio el resto de la gente…………………………………………………………………………..	1	2	3	4	5	6	7
6. Cuando otra gente habla acerca de hacer ejercicio, presto atención para saber si hago tanto ejercicio como ellos hacen……………………………………………	1	2	3	4	5	6	7
7. Comparo lo que elijo comer con los que elige el resto de la gente…………….	1	2	3	4	5	6	7
8. Me fijo en si los alimentos que tomo son más o menos saludables que los que toman los demás………………………………………………………………....	1	2	3	4	5	6	7
9. Comparo partes específicas de mi cuerpo (e.g., estómago, caderas, pecho, etc.) con las de otras personas…………………………………………………….....	1	2	3	4	5	6	7
10. Cuando hago ejercicio junto con otra gente pienso en cuántas calorías estoy quemando en comparación con las que queman ellos…………………………...	1	2	3	4	5	6	7
11. Cuando como con otras personas comparo la cantidad de alimentos que tomo con la que toman ellos…………………………………………………………..	1	2	3	4	5	6	7
12. Comparo la forma de mi cuerpo con la de otras personas…………………….	1	2	3	4	5	6	7
13. Cuando veo a alguien que viste ropas que dejan al descubierto partes del cuerpo, comparo su cuerpo con el mío……………………………………………….	1	2	3	4	5	6	7
14. Comparo la frecuencia con la que hacen ejercicio otras personas con la frecuencia con la que yo lo hago………………………………………………………	1	2	3	4	5	6	7
15. Cuando hago ejercicio junto a otra gente, me fijo en la intensidad a la que lo hacen……………………………………………………………………………………..	1	2	3	4	5	6	7
16. Comparo la cantidad de comida basura que toma la gente que me rodea con la que tomo yo……………………………………………………………………...	1	2	3	4	5	6	7
17. Presto atención a si mi cuerpo está igual de tonificado que el de la gente que me rodea…………………………………………………………………………...	1	2	3	4	5	6	7
18. Comparo la intensidad a la que hago ejercicio con la intensidad a la que lo hace el resto de la gente……………………………………………………………...	1	2	3	4	5	6	7
