# Supplementary material for: Psychometric Examination of the Body, Eating, and Exercise Comparison Orientation Measure (BEECOM) among Spanish Adolescents and Young Adults
Source: Nutrients. 2023 Jan 26;15(3):626. doi: 10.3390/nu15030626 (PMC9921885; doi:10.3390/nu15030626)
Supplement: Supplementary file 1 [file nutrients-15-00626-s001.zip › Tables S1-S3.pdf]

Psychometric Examination of the Body, Eating, and Exercise Comparison Orientation Measure (BEECOM) among Spanish Adolescents and Young Adults

- Table S1: Results of moderation analyses examining the conditional effects of BEECOM scores on overall eating disorders symptoms
- Table S2: Results of moderation analyses examining the conditional effects of BEECOM scores on weight and shape concerns
- Table S3: Results of moderation analyses examining the conditional effects of BEECOM scores on dietary restraint

Table S1

Results of Moderation Analyses Examining the Conditional Effects of BEECOM Scores on Overall Eating Disorders Symptoms

	BEECOM-L								BEECOM-S							
							Interaction								Interaction	
Independent variables	*β* (*SE*)	*t*	*p*	LLCI	ULCI	R^2	ΔR^2	*p*	*β* (*SE*)	*t*	*p*	LLCI	ULCI	R^2	ΔR^2	*p*
Model 1						**.451**	**.005**	**.046**						.438	.004	.074
Constant	**.723 (.026)**	**27.906**	**< .001**	**.673**	**.774**				**.724 (.026)**	**27.360**	**< .001**	**.672**	**.776**			
Percentile-BMI	**.128 (.013)**	**10.036**	**< .001**	**.103**	**.154**				**.131 (.013)**	**10.161**	**< .001**	**.106**	**.157**			
PACS-R	**.044 (.021)**	**2.148**	**.032**	**.004**	**.084**				**.053 (.021)**	**2.547**	**.011**	**.012**	**.094**			
BEECOM (Total)	**.375 (.032)**	**11.802**	**< .001**	**.313**	**.437**				**.362 (.033)**	**11.049**	**< .001**	**.298**	**.426**			
Younger males vs Younger females (D_1)	**-.115 (.040)**	**-2.858**	**.004**	**-.194**	**-.036**				**-.125 (.041)**	**-3.092**	**.002**	**-.205**	**-.046**			
Older females vs Younger females (D_2)	**-.137 (.033)**	**-4.122**	**< .001**	**-.202**	**-.072**				**-.131 (.034)**	**-3.876**	**< .001**	**-.197**	**-.065**			
Older males vs Younger females (D_3)	**-.188 (.036)**	**-5.164**	**< .001**	**-.259**	**-.116**				**-.192 (.037)**	**-5.237**	**< .001**	**-.264**	**-.120**			
BEECOM (Total) x D_1	-.047 (.042)	-1.102	.271	-.129	.036				-.065 (.042)	-1.554	.121	-.147	.017			
BEECOM (Total) x D_2	**-.095 (-037)**	**-2.555**	**.011**	**-168**	**-.022**				**-.089 (.038)**	**-2.365**	**.018**	**-.163**	**-.015**			
BEECOM (Total) x D_3	**-.100 (.047)**	**-2.129**	**.033**	**-.191**	**-.008**				**-.099 (.049)**	**-2.028**	**.043**	**-.195**	**-.003**			
Model 2						.456	.004	.065						.444	.003	.159
Constant	**.711 (.025)**	**27.980**	**< .001**	**.661**	**.760**				**.710 (.026)**	**27.566**	**< .001**	**.660**	**.761**			
Percentile-BMI	**.127 (.013)**	**9.775**	**< .001**	**.101**	**.152**				**.132 (.013)**	**10.162**	**< .001**	**.106**	**.157**			
PACS-R	.031 (.022)	1.418	.156	-.012	.074				.042 (.021)	1.934	.053	-.001	.084			
BEECOM (Body)	**.232 (.034)**	**6.916**	**< .001**	**.166**	**.298**				**.212 (.032)**	**6.541**	**< .001**	**.148**	**.275**			
BEECOM (Eating)	**.136 (.024)**	**5.577**	**< .001**	**.088**	**.184**				**.153 (.022)**	**.6.833**	**< .001**	**.109**	**.197**			
BEECOM (Exercise)	-044 (.023)	1.911	.056	-.001	.090				.030 (.020)	1.538	.124	-.008	.069			
Younger males vs Younger females (D_1)	**-.084 (.041)**	**-2.043**	**.041**	**-.165**	**-.003**				**-.102 (.041)**	**-2.510**	**.012**	**-.182**	**-.022**			
Older females vs Younger females (D_2)	**-.136 (.033)**	**-4.132**	**< .001**	**-.200**	**-.071**				**-.127 (.033)**	**-3.848**	**< .001**	**-.192**	**-.062**			
Older males vs Younger females (D_3)	**-.163 (.037)**	**-4.400**	**< .001**	**-.236**	**-.090**				**-.165 (.037)**	**-4.439**	**< .001**	**-.239**	**-.092**			
BEECOM (Body) x D_1	-.032 (.043)	-0.744	.457	-.117	.053				-.038 (.042)	-0.908	.364	-.121	.044			
BEECOM (Body) x D_2	**-.092 (.035)**	**-2.633**	**.009**	**-.160**	**-.023**				**-.080 (.036)**	**-2.233**	**.026**	**-.150**	**-.010**			
BEECOM (Body) x D_3	-.062 (.043)	-1.427	.154	-.146	0.23				-.058 (.045)	-1.311	.190	-.146	.029			
Model 3						**.458**	**.006**	**.043**						.446	.005	.076
Constant	**.718 (.027)**	**26.933**	**< .001**	**.666**	**.771**				**.719 (.027)**	**26.671**	**< .001**	**.666**	**.772**			
Percentile-BMI	**.127 (.013)**	**9.805**	**< .001**	**.101**	**.152**				**.131 (.013)**	**10.168**	**< .001**	**.106**	**.157**			

PACS-R	.029 (.022)	1.308	.191	-.014	.071				.038 (.022)	1.782	.075	-.004	.081			
BEECOM (Body)	**.178 (.025)**	**7.003**	**< .001**	**.128**	**.228**				**.165 (.023)**	**7.323**	**< .001**	**.121**	**.210**			
BEECOM (Eating)	**.191 (.036)**	**5.272**	**< .001**	**.120**	**.262**				**.218 (.036)**	**6.028**	**< .001**	**.147**	**.289**			
BEECOM (Exercise)	**.045 (.023)**	**1.982**	**.048**	**.000**	**.090**				.030 (.020)	1.552	.121	-.008	.069			
Younger males vs Younger females (D_1)	**-.088 (.0419**	**-2.155**	**.031**	**-.169**	**-.008**				**-.110 (.040)**	**-2.748**	**.006**	**-.189**	**-.032**			
Older females vs Younger females (D_2)	**-.148 (.034)**	**-4.330**	**< .001**	**-.215**	**-.081**				**-.139 (.034)**	**-4.022**	**< .001**	**-.206**	**-.071**			
Older males vs Younger females (D_3)	**-.168 (.037)**	**-4.580**	**< .001**	**-.240**	**-.096**				**-.171 (-037)**	**-4.632**	**< .001**	**-.244**	**-.099**			
BEECOM (Eating) x D_1	-.010 (.047)	-0.202	.840	-.102	.083				-.050 (.046)	-1.105	.270	-.140	.039			
BEECOM (Eating) x D_2	**-.086 (.039)**	**-2.208**	**.027**	**-.162**	**-.010**				**-.098 (.040)**	**-2.437**	**.015**	**-.176**	**-.019**			
BEECOM (Eating) x D_3	**-.103 (.046)**	**-2.212**	**.027**	**-.194**	**-.012**				**-.096 (.049)**	**-1.958**	**.051**	**-.193**	**.000**			
Model 4						.456	.004	.090						.446	.004	.071
Constant	**.720 (.027)**	**27.082**	**< .001**	**.668**	**.772**				**.720 (.027)**	**26.930**	**< .001**	**.668**	**.773**			
Percentile-BMI	**.126 (.013)**	**9.730**	**< .001**	**.100**	**.151**				**.130 (.013)**	**10.039**	**< .001**	**.105**	**.155**			
PACS-R	.028 (.022)	1.268	.205	-.015	.070				.038 (.022)	1.788	.074	-.004	.081			
BEECOM (Body)	**.178 (.026)**	**6.956**	**< .001**	**.128**	**.228**				**.164 (.023)**	**7.294**	**< .001**	**.120**	**.209**			
BEECOM (Eating)	**.135 (.024)**	**5.516**	**< .001**	**.087**	**.182**				**.151 (.022)**	**6.817**	**< .001**	**.108**	**.195**			
BEECOM (Exercise)	**.113 (.036)**	**3.147**	**.002**	**.042**	**.183**				**.100 (.032)**	**3.108**	**.002**	**.037**	**.163**			
Younger males vs Younger females (D_1)	**-.100 (.039)**	**-2.595**	**.010**	**-.176**	**-.024**				**-.117 (.039)**	**-3.000**	**.003**	**-.193**	**-.040**			
Older females vs Younger females (D_2)	**-.155 (.035)**	**-4.444**	**< .001**	**-.223**	**-.087**				**-.145 (.035)**	**-4.147**	**< .001**	**-.214**	**-.077**			
Older males vs Younger females (D_3)	**-.169 (.036)**	**-4.661**	**< .001**	**.241**	**-.098**				**-.170 (.036)**	**4.702**	**< .001**	**-.241**	**-.099**			
BEECOM (Exercise) x D_1	-.059 (.0419	-1.413	.158	-.140	.023				**-.086 (.040)**	**-2.119**	**.034**	**-.165**	**-.006**			
BEECOM (Exercise) x D_2	**-.093 (.040)**	**-2.327**	**.020**	**-.172**	**-.015**				**-.082 (.039)**	**-2.117**	**.034**	**-.157**	**-.006**			
BEECOM (Exercise) x D_3	**-.092 (.045)**	**-2.032**	**.042**	**-.181**	**-.003**				**-.095 (.044)**	**-2.172**	**.030**	**-.180**	**-.009**			

Note. β = Standardized regression coefficients; *SE* = Standard error; CI = Confidence interval; R^2 = Explained Variance; ED = Eating Disorders; ΔR^2 = R^2 increase due to interaction; BEECOM = Body, Eating, and Exercise Comparison Orientation Measure (-L = long version, -S = short version); LLCI, lower limit 95% bootstrap confidence interval; ULCI, upper limit 95% bootstrap confidence interval; PACS-R = Physical Appearance Comparison Scale-Revised.

Table S2

Results of Moderation Analyses Examining the Conditional Effects of BEECOM Scores on Weight and Shape Concerns

Independent variables	BEECOM-L								BEECOM-S							
	β (*SE*)	*t*	*p*	LLCI	ULCI	R^2	Interaction		β (*SE*)	*t*	*p*	LLCI	ULCI	R^2	Interaction	
							ΔR^2	*p*							ΔR^2	*p*
Model 1						**.407**	**.007**	**.009**						**.396**	**.006**	**.015**
Constant	**.933 (.040)**	**23.124**	**< .001**	**.854**	**1.012**				**.933 (.041)**	**22.904**	**< .001**	**.853**	**1.013**			
Percentile-BMI	**.208 (.019)**	**10.765**	**< .001**	**.170**	**.246**				**.211 (.019)**	**10.866**	**< .001**	**.173**	**.249**			
PACS-R	**.077 (.030)**	**2.559**	**.011**	**.018**	**.135**				**.090 (.030)**	**2.966**	**.003**	**.030**	**.149**			
BEECOM (Total)	**.469 (.045)**	**10.497**	**< .001**	**.381**	**.556**				**.455 (.046)**	**9.873**	**< .001**	**.365**	**.546**			
Younger males vs Younger females (D_1)	**-.280 (.058)**	**-4.839**	**< .001**	**-.393**	**-.166**				**-.295 (.058)**	**-5.053**	**< .001**	**-.409**	**-.180**			
Older females vs Younger females (D_2)	**-.145 (.052)**	**-2.785**	**.005**	**-.247**	**-.043**				**-.137 (.053)**	**-2.605**	**.009**	**-.240**	**-.034**			
Older males vs Younger females (D_3)	**-.353 (.055)**	**-6.441**	**< .001**	**-.461**	**-.246**				**-.357 (.055)**	**-6.489**	**< .001**	**-.466**	**-.249**			
BEECOM (Total) x D_1	**-.072 (.056)**	**-1.285**	**.199**	**-.183**	**.038**				**-.108 (.057)**	**-1.903**	**.057**	**-.219**	**.003**			
BEECOM (Total) x D_2	**-.114 (.051)**	**-2.239**	**.025**	**-.214**	**-.014**				**-.114 (.052)**	**-2.197**	**.028**	**-.216**	**-.012**			
BEECOM (Total) x D_3	**-.208 (.064)**	**-.3233**	**.001**	**-.334**	**-.082**				**-.203 (.067)**	**-3.052**	**.002**	**-.334**	**-.073**			
Model 2						**.415**	**.006**	**.010**						**.405**	**.005**	**.034**
Constant	**.909 (.039)**	**23.098**	**< .001**	**.831**	**.986**				**.908 (.040)**	**22.889**	**< .001**	**.830**	**.986**			
Percentile-BMI	**.204 (.019)**	**10.505**	**< .001**	**.166**	**.242**				**.211 (.019)**	**10.829**	**< .001**	**.172**	**.249**			
PACS-R	.050 (.031)	1.598	.110	-.011	.111				**.067 (.031)**	**2.179**	**.030**	**.007**	**.127**			
BEECOM (Body)	**.361 (.049)**	**7.407**	**< .001**	**.265**	**.456**				**.331 (.046)**	**7.173**	**< .001**	**.241**	**.422**			
BEECOM (Eating)	**.142 (.035)**	**4.064**	**< .001**	**.074**	**.211**				**.168 (.032)**	**5.222**	**< .001**	**.105**	**.231**			
BEECOM (Exercise)	.033 (.034)	0.975	.330	-.034	.100				.015 (.029)	0.529	.597	-.041	.072			
Younger males vs Younger females (D_1)	**-.221 (.060)**	**-3.682**	**< .001**	**-.338**	**-.103**				**-.250 (.059)**	**-4.237**	**< .001**	**-.366**	**-.134**			
Older females vs Younger females (D_2)	**-.136 (.051)**	**-2.655**	**.008**	**-.237**	**-.036**				**-.126 (.052)**	**-2.433**	**.015**	**-.227**	**-.024**			
Older males vs Younger females (D_3)	**-.314 (.056)**	**-5.618**	**< .001**	**-.423**	**-.204**				**-.315 (.056)**	**-5.627**	**< .001**	**-.425**	**-.205**			
BEECOM (Body) x D_1	-.048 (.062)	-0.781	.435	-.169	.073				-.079 (.060)	-1.309	.191	-.198	.040			
BEECOM (Body) x D_2	**-.148 (.051)**	**-2.926**	**.003**	**-.247**	**-.049**				**-.136 (.052)**	**-2.631**	**.009**	**-.237**	**-.035**			
BEECOM (Body) x D_3	**-.161 (.064)**	**-2.529**	**.012**	**-.285**	**-.036**				**-.149 (.064)**	**-2.304**	**.021**	**-.275**	**-.022**			
Model 3						**.416**	**.007**	**.009**						**.406**	**.006**	**.036**
Constant	**.920 (.041)**	**22.632**	**< .001**	**.841**	**1.000**				**.922 (.041)**	**22.505**	**< .001**	**.842**	**1.002**			
Percentile-BMI	**.203 (.019)**	**10.472**	**< .001**	**.165**	**.241**				**.209 (.109)**	**10.752**	**< .001**	**.171**	**.247**			

PACS-R	.042 (.031)	1.330	.184	-.020	.103				.059 (.031)	1.911	.056	-.002	.120			
BEECOM (Body)	**.261 (.039)**	**6.770**	**< .001**	**.185**	**.337**				**.238 (.034)**	**7.034**	**< .001**	**.171**	**.304**			
BEECOM (Eating)	.040 (.034)	1.190	.234	-.026	.106				.018 (.029)	0.633	.527	-.038	.075			
BEECOM (Exercise)	**.216 (.052)**	**4.161**	**< .001**	**.114**	**.317**				**.263 (.051)**	**5.126**	**< .001**	**.162**	**.363**			
Younger males vs Younger females (D_1)	**-.237 (.058)**	**-4.074**	**< .001**	**-.351**	**-.123**				**-.268 (.058)**	**-4.643**	**< .001**	**-.381**	**-.155**			
Older females vs Younger females (D_2)	**-.164 (.053)**	**-3.090**	**.002**	**-.268**	**-.060**				**-.150 (.053)**	**-2.819**	**.005**	**-.254**	**-.046**			
Older males vs Younger females (D_3)	**-.313 (.055)**	**-5.716**	**< .001**	**-.421**	**-.206**				**-.319 (.055)**	**-5.772**	**< .001**	**-.427**	**-.210**			
BEECOM (Eating) x D_1	-.022 (.061)	-0.358	.720	-.141	.097				-.091 (.061)	-1.498	.134	-.211	.028			
BEECOM (Eating) x D_2	-.070 (.054)	-1.290	.197	-.176	.036				-.099 (.061)	-1.769	.077	-.209	.011			
BEECOM (Eating) x D_3	**-.204 (.064)**	**-3.191**	**.001**	**-.329**	**-.078**				**-.193 (.067)**	**-2.899**	**.004**	**-.324**	**-.062**			
Model 4						.413	.004	.065						**.405**	**.004**	**.044**
Constant	**.923 (.040)**	**22.928**	**< .001**	**.844**	**1.002**				**.923 (.040)**	**22.815**	**< .001**	**.844**	**1.003**			
Percentile-BMI	**.203 (-019)**	**10.445**	**< .001**	**.165**	**.241**				**.209 (.019)**	**10.750**	**< .001**	**.170**	**.247**			
PACS-R	.042 (.031)	1.350	.177	-.019	.104				**.061 (.031)**	**1.988**	**.047**	**.001**	**.122**			
BEECOM (Body)	**.263 (.039)**	**6.796**	**< .001**	.187	.339				**.238 (.034)**	**7.067**	**< .001**	**.172**	**.304**			
BEECOM (Eating)	**.141 (.035)**	**4.028**	**< .001**	.072	.210				**.165 (.032)**	**5.210**	**< .001**	**.103**	**.228**			
BEECOM (Exercise)	**.129 (.050)**	**2.564**	**.010**	.030	.227				**.112 (.045)**	**2.455**	**.014**	**.022**	**.201**			
Younger males vs Younger females (D_1)	**-.250 (-056)**	**-4.491**	**< .001**	-.360	-.141				**-.272 (.056)**	**-4.846**	**< .001**	**-.383**	**-.162**			
Older females vs Younger females (D_2)	**-.165 (.053)**	**-3.090**	**.002**	-.270	-.060				**.152 (.054)**	**-2.845**	**.005**	**-.258**	**-.047**			
Older males vs Younger females (D_3)	**-.313 (.054)**	**-5.748**	**< .001**	-.420	-.206				**-.313 (.054)**	**-5.761**	**< .001**	**-.420**	**-.206**			
BEECOM (Exercise) x D_1	-.082 (.055)	-1.506	.132	-.190	.025				**-.114 (.055)**	**-2.097**	**.036**	**-.222**	**-.007**			
BEECOM (Exercise) x D_2	-.103 (.054)	-1.912	.056	-.208	.003				-.094 (.053)	-1.777	.076	-.198	.010			
BEECOM (Exercise) x D_3	**-.159 (.062)**	**-2.571**	**.010**	**-.280**	**-.038**				**-.160 (.060)**	**-2.662**	**.008**	**-.279**	**-.042**			

Note. β = Standardized regression coefficients; *SE* = Standard error; CI = Confidence interval; R^2 = Explained Variance; ΔR^2 = R^2 increase due to interaction; ED = Eating Disorders; BEECOM = Body, Eating, and Exercise Comparison Orientation Measure (-L = long version, -S = short version); LLCI, lower limit 95% bootstrap confidence interval; ULCI, upper limit 95% bootstrap confidence interval; PACS-R = Physical Appearance Comparison Scale-Revised.

Table S3

Results of Moderation Analyses Examining the Conditional Effects of BEECOM Scores on Dietary Restraint

	BEECOM-L								BEECOM-S							
Independent variables	*β* (*SE*)	*t*	*p*	LLCI	ULCI	R^2	Interaction		*β* (*SE*)	*t*	*p*	LLCI	ULCI	R^2	Interaction	
							ΔR^2	*p*							ΔR^2	*p*
Model 1						**.279**	.006	.061						.272	.005	.116
Constant	**.692 (.039)**	**17.738**	**< .001**	**.616**	**.769**				**.692 (.040)**	**17.394**	**< .001**	**.614**	**.770**			
Percentile-BMI	**.214 (.019)**	**11.181**	**< .001**	**.177**	**.252**				**.217 (.019)**	**11.320**	**< .001**	**.180**	**.255**			
PACS-R	-.007 (.028)	-0.235	.815	-.062	.048				.001 (.028)	0.034	.973	-.055	.056			
BEECOM (Total)	**.397 (.047)**	**8.403**	**< .001**	**.304**	**.490**				**.375 (.049)**	**7.693**	**< .001**	**.280**	**.471**			
Younger males vs Younger females (D_1)	**-.135 (.056)**	**-2.400**	**.017**	**-.245**	**-.025**				**-.141 (.057)**	**-.2485**	**.013**	**-.251**	**-.030**			
Older females vs Younger females (D_2)	-.078 (.050)	-1.561	.119	-.177	.020				-.072 (.051)	-1.426	.154	-.172	.027			
Older males vs Younger females (D_3)	**-.153 (.054)**	**-2.847**	**.004**	**-.259**	**-.048**				**-.157 (.054)**	**-2.894**	**.004**	**-.263**	**-.051**			
BEECOM (Total) x D_1	**-.137 (.059)**	**-2.302**	**.022**	**-.253**	**-.020**				**-.131 (.061)**	**-2.152**	**.032**	**-.250**	**-.012**			
BEECOM (Total) x D_2	**-.120 (.054)**	**-2.229**	**.026**	**-.225**	**-.014**				-.101 (.055)	-1.840	.066	-.209	.007			
BEECOM (Total) x D_3	**-.139 (.065)**	**-2.122**	**.034**	**-.267**	**-.010**				-.129 (.067)	-1.919	.055	-.260	.003			
Model 2						**.284**	**.006**	**.042**						.279	.004	.134
Constant	**.690 (.038)**	**18.019**	**< .001**	**.615**	**.765**				**.690 (.039)**	**17.818**	**< .001**	**.614**	**.766**			
Percentile-BMI	**.220 (.019)**	**11.379**	**< .001**	**.182**	**.258**				**.225 (.019)**	**11.700**	**< .001**	**.187**	**.262**			
PACS-R	.017 (.030)	0.577	.564	-.041	.075				.021 (.029)	0.723	.470	-.036	.078			
BEECOM (Body)	**.142 (.048)**	**2.959**	**.003**	**.048**	**.236**				**.120 (.048)**	**2.516**	**.012**	**.026**	**.214**			
BEECOM (Eating)	**.190 (.035)**	**5.362**	**< .001**	**.120**	**.259**				**.204 (.033)**	**6.139**	**< .001**	**.139**	**.269**			
BEECOM (Exercise)	.073 (.032)	2.271	.023	.010	.137				.060 (.029)	2.053	.040	.003	.118			
Younger males vs Younger females (D_1)	**-.142 (.057)**	**-2.506**	**.012**	**-.253**	**-.031**				**-.152 (-.056)**	**-2.696**	**.007**	**-.263**	**-.041**			
Older females vs Younger females (D_2)	-.070 (.050)	-1.409	.159	-.168	.028				-.064 (.050)	-1.278	.201	-.162	.034			
Older males vs Younger females (D_3)	-.160 (.054)	-2.933	.003	-.266	-.053				-.161 (.054)	-2.975	.003	-.267	-.055			
BEECOM (Body) x D_1	**-.147 (.060)**	**-2.465**	**.014**	**-.265**	**-.030**				**-.126 (.059)**	**-2.132**	**.033**	**-.243**	**-.010**			
BEECOM (Body) x D_2	**-.124 (.050)**	**-2.500**	**.013**	**-.222**	**-.027**				**-.102 (.051)**	**-1.998**	**.046**	**-.203**	**-.002**			
BEECOM (Body) x D_3	-.099 (.064)	-1.552	.121	-.224	.026				-.084 (.064)	-1.320	.187	-.209	.041			
Model 3																
Constant	**.703 (.040)**	**17.553**	**< .001**	**.624**	**.782**	**.285**	**.006**	**.008**	**.702 (.040)**	**17.403**	**< .001**	**.623**	**.781**	.281	.006	.104
Percentile-BMI	**.219 (.019)**	**11.376**	**< .001**	**.181**	**.257**				**.223 (.019)**	**11.680**	**< .001**	**.186**	**.261**			

PACS-R	.013 (.030)	0.457	.648	-.044	.071				.017 (.029)	0.591	.555	-.040	.074			
BEECOM (Body)	.048 (.036)	1.335	.182	-.022	.118				.041 (.033)	1.268	.205	-.023	.105			
BEECOM (Eating)	**.282 (.054)**	**5.258**	**< .001**	**.177**	**.388**				**.298 (.053)**	**5.590**	**< .001**	**.193**	**.402**			
BEECOM (Exercise)	**.073 (.032)**	**2.261**	**.024**	**.010**	**.137**				**.060 (.029)**	**2.028**	**.043**	**.002**	**.118**			
Younger males vs Younger females (D_1)	**-.141 (.058)**	**-2.446**	**.015**	**-.254**	**-.028**				**-.155 (.057)**	**-2.711**	**.007**	**-.267**	**-.043**			
Older females vs Younger females (D_2)	-.090 (.051)	-1.774	.076	-.190	.010				-.082 (.051)	-1.599	.110	-.182	.019			
Older males vs Younger females (D_3)	**-.169 (.055)**	**-3.094**	**.002**	**-.276**	**-.062**				**-.171 (.054)**	**-3.131**	**.002**	**-.277**	**-.064**			
BEECOM (Eating) x D_1	-.101 (.063)	-1.595	.111	-.225	.023				-.116 (.065)	-1.789	.074	-.244	.011			
BEECOM (Eating) x D_2	-.102 (.057)	-1.792	.073	-.213	.010				-.099 (.058)	-1.706	.088	-.212	.015			
BEECOM (Eating) x D_3	**-.173 (.068)**	**-2.535**	**.011**	**-.308**	**-.039**				**-.167 (.071)**	**-2.355**	**.019**	**-.306**	**-.028**			
Model 4						.283	.005	.114						.279	.004	.160
Constant	**.704 (.040)**	**17.603**	**< .001**	**.626**	**.782**				**.702 (.040)**	**17.421**	**< .001**	**.623**	**.781**			
Percentile-BMI	**.218 (.019)**	**11.298**	**< .001**	**.180**	**.256**				**.223 (.019)**	**11.602**	**< .001**	**.185**	**.260**			
PACS-R	.015 (.030)	0.509	.611	-.043	.073				.021 (.029)	0.704	.482	-.037	.078			
BEECOM (Body)	.049 (.036)	1.361	.174	-.022	.119				.042 (.032)	1.304	.192	-.021	.106			
BEECOM (Eating)	**.187 (.035)**	**5.329**	**< .001**	**.118**	**.256**				**.200 (.033)**	**6.092**	**< .001**	**.136**	**.265**			
BEECOM (Exercise)	**.168 (.051)**	**3.275**	**.001**	**.067**	**.269**				**.143 (.048)**	**2.979**	**.003**	**.049**	**.237**			
Younger males vs Younger females (D_1)	**-.142 (.056)**	**-2.546**	**.011**	**-.252**	**-.033**				**-.156 (.056)**	**-2.777**	**.006**	**-.266**	**-.046**			
Older females vs Younger females (D_2)	-.092 (.052)	-1.781	.075	-.193	.009				-.082 (.052)	-1.593	.112	-.184	.019			
Older males vs Younger females (D_3)	**-.170 (.054)**	**-3.132**	**.002**	**-.276**	**-.064**				**-.170 (.054)**	**-3.165**	**.002**	**-.275**	**-.065**			
BEECOM (Exercise) x D_1	**-.127 (.059)**	**-2.160**	**.031**	**-.242**	**-.012**				**-.127 (.060)**	**-2.126**	**.034**	**-.244**	**-.010**			
BEECOM (Exercise) x D_2	**-.114 (.056)**	**-2.034**	**.042**	**-.225**	**-.004**				-.092 (.056)	-1.656	.098	-.202	.017			
BEECOM (Exercise) x D_3	-.119 (.063)	-1.877	.061	-.243	.005				-.100 (.065)	-1.551	.121	-.227	.027			

Note. β = Standardized regression coefficients; *SE* = Standard error; CI = Confidence interval; R^2 = Explained Variance; ΔR^2 = R^2 increase due to interaction; ED = Eating Disorders; BEECOM = Body, Eating, and Exercise Comparison Orientation Measure (-L = long version, -S = short version); LLCI, lower limit 95% bootstrap confidence interval; ULCI, upper limit 95% bootstrap confidence interval; PACS-R = Physical Appearance Comparison Scale-Revised.
